# Protection of Animals during Transport: Analysis of the Infringements Reported from 2009 to 2013 during On-Road Inspections in Italy

**DOI:** 10.3390/ani10020356

**Published:** 2020-02-22

**Authors:** Barbara Padalino, Roberta Barrasso, Daniele Tullio, Martina Zappaterra, Leonardo Nanni Costa, Giancarlo Bozzo

**Affiliations:** 1 Department of Agricultural and Food Sciences, University of Bologna, Viale Giuseppe Fanin 46, 40127 Bologna, Italyleonardo.nannicosta@unibo.it (L.N.C.); 2 Department of Veterinary Medicine, University of Bari, Strada Provinciale per Casamassima km 3, 70010 Valenzano, Italy; Roberta.barrasso@uniba.it (R.B.); Giancarlo.bozzo@uniba.it (G.B.); 3 ASL BA-Local Health Authority Veterinary Service, Via dei Mille 29, 70126 Bari, Italy; Daniele.tullio@asl.bari.it

**Keywords:** transportation, welfare, law, infringement, document, vehicle

## Abstract

**Simple Summary:**

On-road inspections of vehicles that transport animals are mandatory in Europe. Infringements of the Council Regulation (EC) 1/2005 that were ascertained by competent authorities during on-road inspections published by the Italian Health Ministry from 2009 to 2013 were analyzed. The aims were both to identify possible routes or species that are more likely to be at risk of poor welfare conditions and to suggest recommendations. A total of 985 infringements were reported. For analysis, they were split into three main categories that were related to animal welfare (AW), vehicle (V), and accompanying documents (D). Each category was further classified under different subcategories (e.g., overcrowding for AW, lack of drinking system for V, and lack of health certificate for D). The most frequent infringements were related to D (34.4%), but more than one infringement was often found during an inspection (mean: 1.58; max: 9). A score (from 1 to 3) that was related to the severity of the animal-welfare issues was created which was found to be associated with year, species, authority, and country of dispatch (*p* < 0.001). Over the years, the only improvement was in the accompanying documentation. Vehicles that were transporting pigs, sheep, or goats were more likely to have the poorest welfare conditions, whilst vehicles that were transporting horses or other species, including dogs, were often found with irregular documentation. AW infringements were more likely to be uncovered during road inspections where traffic police and veterinarians worked together. This type of road inspection should be intensified so as to enhance animal welfare during transportation.

**Abstract:**

Council Regulation (EC) No 1/2005 requires that vehicles that are transporting animals be subjected to checks conducted by competent authorities. Yearly, each member state sends a report to the European government on the infringements that have been discovered during on-road inspections. The reports that were published by the Italian Ministry of Public Health from 2009 to 2013 were analyzed. Possible associations between the type of infringement (related to animal welfare (AW), vehicle (V) and accompanying documents (D)), year, season, transported species, place of inspection, and competent authorities were identified. A total of 985 infringements were analyzed, with some vehicles receiving more than one (mean: 1.58; max: 9). A score (from 1 to 3) that was related to the severity of the infringements was created. In 2009 and 2010, there was a 50% higher probability of encountering penalties of a lower severity (D or V) than in 2011 (*p* < 0.0001). Vehicles that were transporting pigs showed the highest probability of committing animal welfare-related infringements (odds ratio (OR) = 3.85, 95% confidence interval (95%CI) = 1.82–8.76, *p* < 0.0001). Vehicles were four times more likely to suffer animal welfare-related penalties when traffic police worked in synergy with veterinary services (OR = 4.12, 95%CI = 1.70–11.13, *p* = 0.0005). Vehicles that were transporting Equidae and “other species,” including pets, for commercial purposes were more likely to be fined for a lack or incompleteness of the veterinary documents than those transporting cattle (*p* = 0.002 and *p* = 0.004, respectively). This study gives statistical evidence of the implementation of EC 1/2005. The training of transporters and drivers on how to manage transport in an animal welfare-friendly manner and a standardized method on how to conduct road inspections among competent authorities are recommended.

## 1. Introduction

Every day, millions of animals are transported around the world for different reasons, from breeding to meat production [1]. Moreover, the reduced number of small abattoirs and the establishment of large slaughterhouses have led to an increase in the duration of trips for animals [2]. It is well known that long-distance animal transport is an animal-welfare issue because it is considered a stressful event that may lead to health problems and prolonged suffering [3]. Several stress factors could negatively affect animal welfare during road transport [4]. They could be related to the experience and the condition of the animals (the withdrawal of feed and water, the thermal and physical conditions inside the vehicle, overcrowding, the absence of partitions, the mixing of animals) or the journey (driving skills, noise, vibration, road quality, and duration) [5,6]. Therefore, road transport is a multifactorial problem that is characterized by a combination of stressors that are responsible for animal welfare as well as food safety, meat quality, and carcass quality [3,7]. The World Organization for Animal Health (OIE) has identified transportation as one of the most important pre-harvest variables with respect to meat quality, and, consequently, the importance of maintaining good animal welfare during transport has been highlighted [8,9,10]. The OIE has issued a series of recommendations on how to manage animal transportation, inviting each country to issue a specific law on the protection of animals during transport.

Council Regulation (EC) No 1/2005 [11] regulates animal transportation in Europe and contains special requirements for journeys exceeding eight hours, including maximum journey duration, stopping at control posts, and on-road inspections. These inspections represent the only opportunity to monitor transport conditions between the loading and unloading of journeys exceeding eight hours. They are mandatory, performed by competent authorities, and include non-discriminatory inspections on-road of animals, vehicles, and accompanying documents [12]. The transported species have different physiological requirements and needs according to transport conditions, means of transport used, and climatic zones [13]. Previous studies [14,15,16] have shown that after a long transport, one hour of rest was insufficient to restore the physiological conditions of adult sheep and that a 12 h stop was preferable to a three hour one. Horses, by contrast, are not able to restore the loss of weight they undergo during long-distance transportation within 24 h [1]. Consequently, EC 1/2005 has special requirements for each species, in particular regarding vehicle design and maximum journey duration. Competent authorities should double-check different criteria depending on the species that are transported during on-road inspections.

In compliance with Article 27 of EC 1/2005, by 30 June of each year, member states have to send the European Commission an annual report for the previous year that contains the principal inspections and infringements. These inspections must be carried out on an adequate proportion of the animals that are transported each year within each member state. The proportion of inspections shall be increased where it has been established that the provisions of this regulation have been disregarded. In Italy in 2011, a memorandum of understanding (MoU) was signed between the Ministry of the Interior and the Ministry of Public Health that allowed traffic police to call official veterinarians in cases involving animal-welfare issues. This MoU came into force in 2013. In March 2018, the Conference of Presidents of the European Parliament tasked a special committee with preparing a report on the implementation of EC 1/2005, and its main conclusion was that there is a limited amount of literature that is related to welfare conditions during the transport of live animals in the EU [17]. To the best of the authors’ knowledge, there has been no study that has analyzed how the type of infringements have varied over the years, identifying possible risk factors depending on the transported species in Italy. Consequently, the aim of this study was to analyze the type of infringements that were uncovered during on-road inspections as set out in the Italian Health Ministry reports that were written in compliance with Article 27 of EC 1/2005 from 2009 to 2013. Possible associations between the type of infringement (related to animal welfare, vehicle and accompanying documents), year, season, transported species, place of inspection, and competent authorities were identified. The results may be useful to propose recommendations on how to implement on-road inspections in Europe.

## 2. Materials and Methods

### 2.1. Dataset

Italian Health Ministry reports on the infringements uncovered during live animal transportation written in compliance with Article 27 of EC 1/2005 from 2009 to 2013 were used [18]. These reports were published online and are freely available, and the authors also informed the head of animal welfare during transport at the Italian Health Ministry of their use for research purposes.

The period from 2009 to 2013 was chosen for the following reasons: (i) Regulation (EC) No 1/2005 came into force in January 2007; (ii) legislative decree No 151 on the sanctioning provision for violations of EC 1/2005 was issued on the 25th of July 2007; (iii) 2008 was considered as a year of training; and (iv) the report format changed in 2014, and data were no longer comparable.

The infringements that were recorded during the inspections were divided into three categories that were related to the welfare of the animals that were being transported (animal welfare: AW), the vehicle (V), and the accompanying documents (D), as already done in the study by Nanni Costa et al. [12]. Each main category was further divided into subcategories. Table 1 shows the main categories with their respective subcategories, with their definitions and examples taken from the original infringements.

A severity score (from 1 to 3) was created to rank the severity of the animal-welfare issues that were found in each vehicle. Vehicles that were fined for an infringement that were related to vehicle or documents scored 1, vehicles that were fined for an infringement that was related to animal welfare or fined for both V and D scored 2, and vehicles that reported multiple infringements (AW + D, AW + V, AW + V+D) scored 3 (Table 2).

### 2.2. Statistical Analysis

Descriptive statistics of the data were obtained by using an online statistical software (Statulatorbeta®, Sydney, Australia) with year, season, species, the country of dispatch, the country of destination, supervisory body, the place of inspection (place), the categories of infringement (AW, V, D), and their subcategories as categorical variables, while the number of infringements were used as numerical variables.

The infringement severity score (Table 2) was analyzed by using univariate ordinal regression analysis with the score as the outcome and with year, season, species, country of dispatch, supervisory body, and place as the fixed factors. The effect of these variables on the scores for the severity of the infringements was individually estimated for each independent variable with the *polr* function of the *MASS* package in the R environment [19]. Data are expressed as the odds ratio (OR) and the 95% confidence interval (95% CI), which were estimated by using basic functions in the *stats* package, and Wald-test *P*-values were calculated by using the *waldtest* function of the *lmtest* package in the R environment.

In order to identify possible associations, a univariate multifactorial logistic analysis was conducted by using the total number of infringements as the dependent variable and year, species, country of dispatch, supervisory body, and place as the fixed factors. A univariate multifactorial logistic analysis was also conducted by using each subcategory of the infringement as binary dummy dependent variable (1/0) and year, species, the country of dispatch, supervisory body, and the inspection place as the fixed factors. The univariate multifactorial logistic analyses were performed in the R environment [19] with the *glm* function of the *stats* package. Data are expressed as OR, 95%CI and Wald-test *p*-values, all of which were calculated as mentioned above. In order to detect and represent underlying structures in the data set, a multiple correspondence analysis (MCA) was also performed. An MCA is an unsupervised type analysis that identifies patterns in a data set by combining the original variables into new descriptive components (named dimensions). Each dimension is determined by a specific combination of the original variables, which, based on their weight in each dimension, contribute in explaining the total variance that is noticed in the sample. Not all data, but only the infringement subcategories, which were found to be significantly associated in univariate logistic regressions with the animal species, supervisory bodies, and places of inspection were considered together for the MCA. The MCA was performed by using the *factoextra* package, and the results were plotted by using the packages *gplots* and *grDevices* in the R environment [19].

## 3. Results

### 3.1. Descriptive Statistics

A total of 985 infringements were analyzed (Table 3). All the vehicles that were sanctioned were transporting animals for commercial purposes. While the numbers of vehicles that were fined varied across the years, with a peak in 2011, rates per season were similar. The majority of the vehicles were transporting cattle and pigs, while the third most frequent species was Equidae, which were transported in 151 of the fined vehicles; dogs and cats were transported in 22 of the fined vehicles. Vehicles were mainly coming from France, Italy or Spain and heading to Italy. Traffic police working in Northwest Italy (Table 3) issued more than 40% of the penalties.

D-type infringements were the most common, with the AW-type being the second most common (Table 4). More than one infringement was often found during an inspection (mean: 1.58; max: 9), so some vehicles were fined in two or even all three categories.

**Figure 1:**
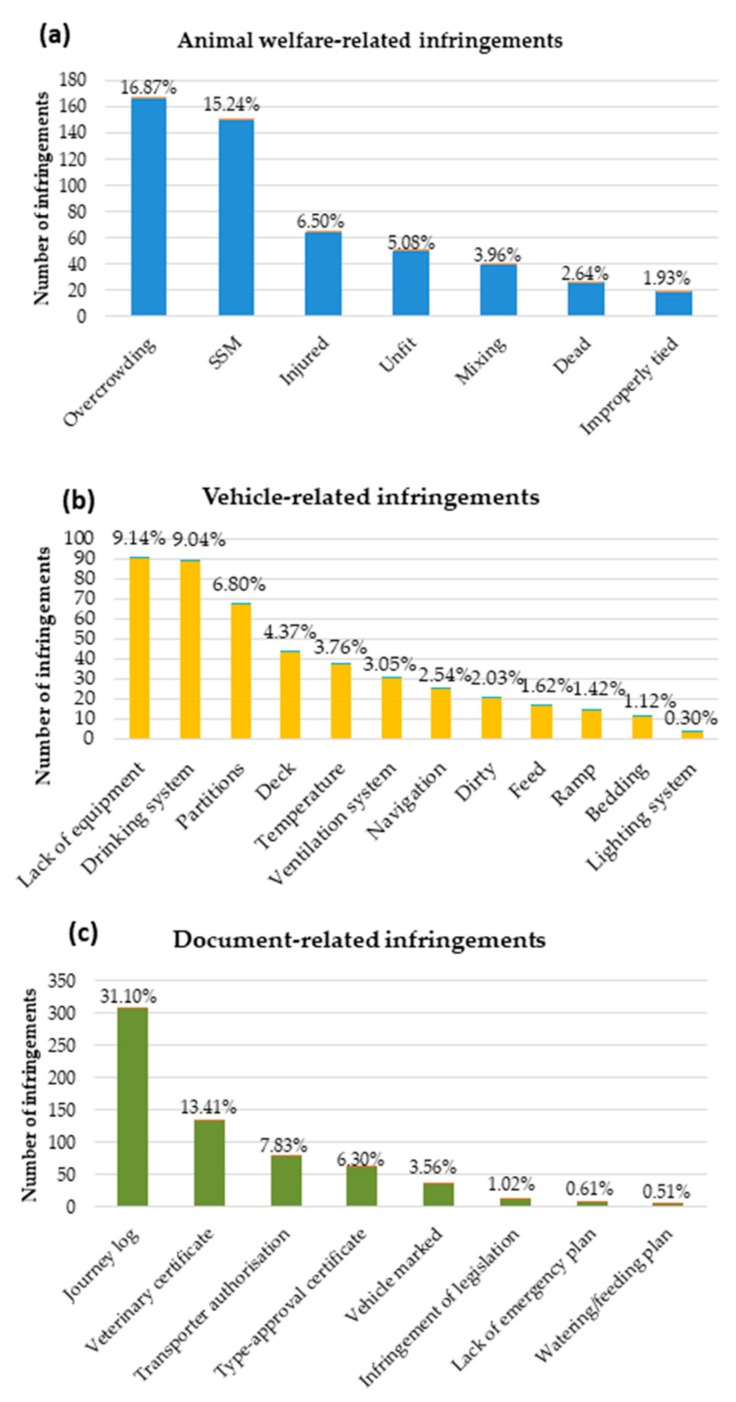
Subcategories of infringements that are related to animal welfare (**a**), vehicle (**b**) and documents (**c**). SSM: scheduled stop missing.

The most common subcategories that were related to AW were overcrowding (16.87%) and the missing of a scheduled stop (SSM; 15.24%; Figure 1a). The most common subcategories that were related to vehicles were a lack of equipment (9.14%) and lack of a drinking system (9.04%) (Figure 1b), while the most common subcategories that were related to documents were journey log (31.10%) and veterinary certificate (13.41%) (Figure 1c).

### 3.2. Univariate Ordinal Regression Analysis

**Figure 2:**
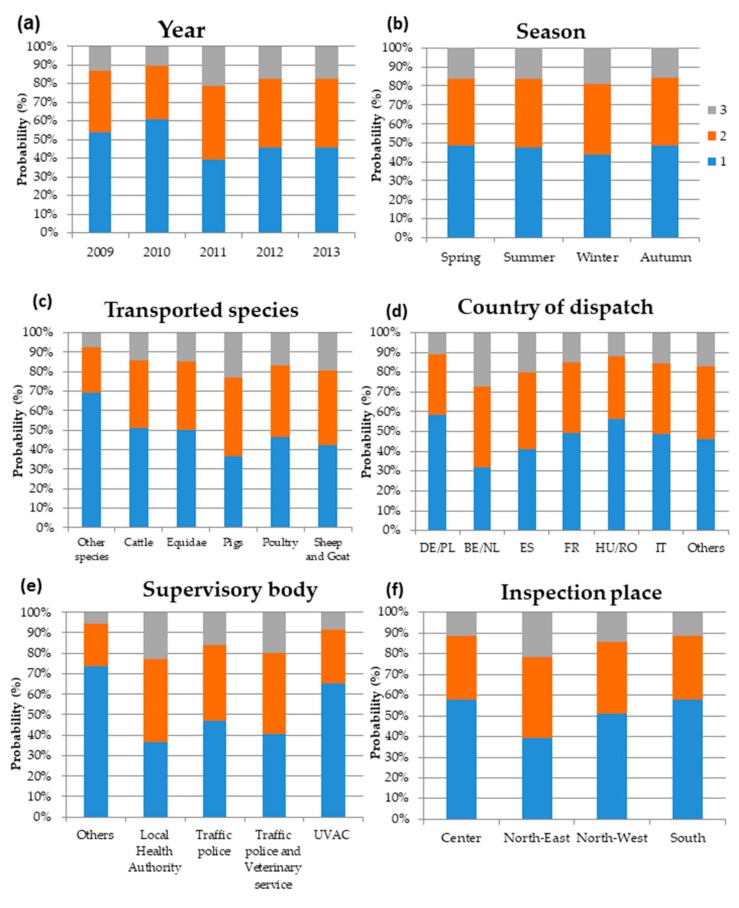
Probability of a more severe infringement being uncovered during on-road inspections (1 (blue) = less severe infringements, 2 (orange) = medium severe infringements, 3 (grey) = the most severe ones) associated with year (**a**), season (**b**), transported animal species (**c**), dispatch country (**d**), supervisory body (**e**), and the place of inspection in Italy (**f**).DE/PL: Germany/Poland; BE/NL: Belgium/the Netherlands; ES: Spain; FR: France; HU/RO: Hungary/Romania; IT: Italy; Other: China; Czech Republic; Denmark; Ireland; Lithuania; Slovakia; Slovenia; United Kingdom; and UVAC: Veterinary Offices for Compliance with EU Requirements.

There was a significant association between ‘year’ and the severity of the infringement that was noticed during on-road inspections (Wald *p* < 0.0001) (Figure 2a). Indeed, vehicles that were fined in 2009 and 2010 had, on average, a 50% lower probability of encountering fines of greater severity than the vehicles that were fined in 2011 (OR = −0.55, 95%CI = from −0.38 to −0.80, *p* = 0.002, for the year 2009 vs. 2011, OR= −0.42, 95%CI = from −0.28 to −0.61, *p* < 0.0001 for the year 2010 vs. 2011). On the other hand, no differences were noticed in the frequency of severe penalties after 2011. Whilst no association was found with ‘seasons’ (Wald *p* = 0.602; Figure 2b), the transported animal ‘species’ showed a strong association with the severity score (Wald *p* = 0.0005). Compared with the “other species” class, vehicles that were transporting cattle, Equidae, pigs, poultry, sheep, and goats were more likely to incur more severe penalties (Figure 2c). Indeed, vehicles that were transporting pigs showed an almost fourfold higher probability of being sanctioned for an animal welfare-related subcategory compared with the “other species” class (OR = 3.85, 95%CI = 1.82–8.76, *p* < 0.0001), and vehicles that were transporting sheep and goats were three times more likely to incur severe penalties (OR = 3.05, 95%CI = 1.38–7.15, *p* = 0.007). The ‘country of dispatch’ also was associated with the severity score (Wald *p* = 0.0005; Figure 2d). The highest probabilities of incurring severe penalties were noticed for vehicles from Belgium/Netherlands and from Spain, proving, respectively, to be three times (OR = 3.01, 95%CI = 1.69–5.43, *p* = 0.0002) and two times (OR = 2.01, 95%CI = 1.14–3.58, *p* = 0.016) more likely to have type 2 and 3 infringements than vehicles from Germany/Poland (Figure 2d).

There was also an association with the different ‘supervisory bodies’ (Wald *p* < 0.0001; Figure 2e). Vehicles were more likely to incur animal welfare-related penalties when inspected by supervisory bodies that were involved in the control of animal health and welfare, such as the local health authority and the veterinary service (Figure 2e). The highest probability of incurring severe penalties was observed for the local health authority (OR = 4.87, 95%CI = 2.10-12.68, *p* = 0.0005) when compared with the “other” supervisory body class. Similarly, when traffic police worked in synergy with the veterinary service, vehicles showed a fourfold increase in the probability of incurring animal welfare-related issues compared with the “others” supervisory body class (OR = 4.12, 95%CI = 1.70–11.13, *p* = 0.0005).

Finally, there was an association with the ‘place’ of inspection; vehicles that were inspected in the northeast of Italy were twice as likely to incur severe penalties as in the center of Italy (OR = 2.16, 95%CI = 1.54–3.03, *p* < 0.0001) (Figure 2f).

### 3.3. Univariate Multifactorial Logistic Regression Analysis

The number of infringements that were noticed for each vehicle were mainly associated with year, the country of dispatch, supervisory body, and, to a lesser extent, with transported animal species. The number of infringements per vehicle in 2011 averaged 1.33, whereas each fined vehicle in 2009 and 2012 averaged almost two infringements (1.81 for 2009, *p*-value of the comparison with 2011 = 0.0002; 1.84 for 2012; *p* of the comparison with 2011 = 0.0008). Compared with fined vehicles that were transporting cattle, those with horses were more likely to incur more than one fine (1.81 for Equidae and 1.55 for cattle, *p* = 0.034). When compared with Italy, all other countries of dispatch were associated with increased probabilities for vehicles to incur more than one fine. Indeed, fined vehicles coming from Germany/Poland were those with the highest probabilities of receiving multiple fines, with an average number of 1.71 against the 1.49 for transporters from Italy (*p* = 0.017). Traffic police were more likely to identify multiple infringements, both when working alone (on average 1.75 infringements discovered, *p* = 0.026) and together with the veterinary service (1.66 infringements, *p* = 0.071) when compared with other supervisory bodies (1.29 infringements).

**Figure 3:**
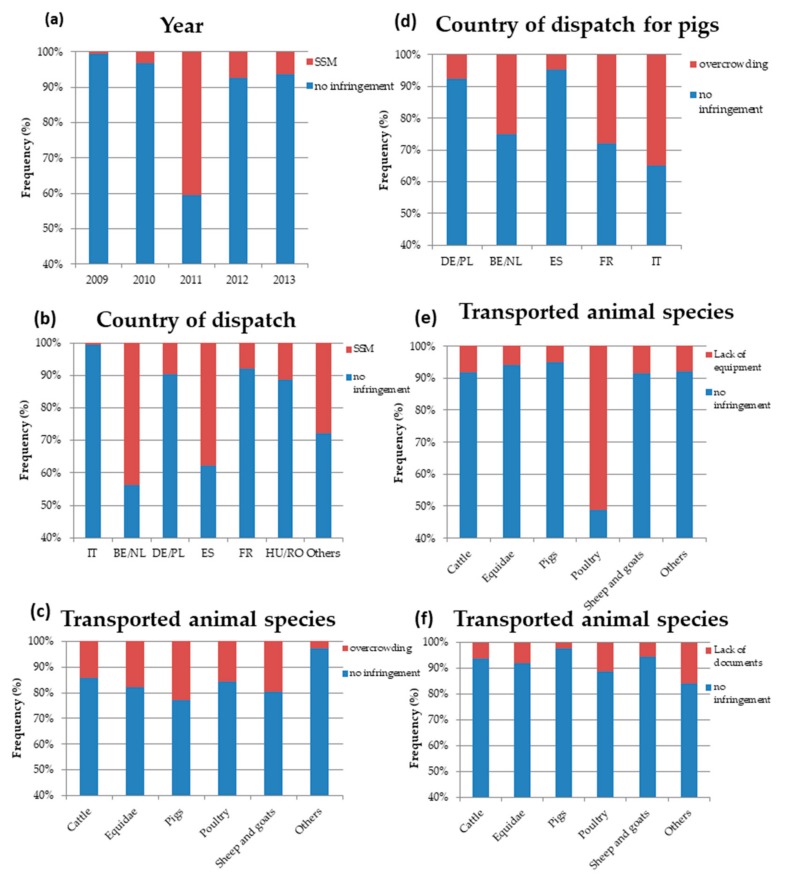
Probability of uncovering infringements due to missing a scheduled stop (SSM) for the considered years (**a**) and for the country of dispatch (**b**). Probability of uncovering infringements due to overcrowding by species (**c**) and by country of dispatch for pigs (**d**). Probability of uncovering infringements due to lack of equipment by species (**e**). Probability of uncovering infringements due to lack of documents by species (**e**). DE/PL: Germany/Poland; BE/NL: Belgium/the Netherlands; ES: Spain; FR: France; HU/RO: Hungary/Romania; IT: Italy; Other: China; Czech Republic; Denmark; Ireland; Lithuania; Slovakia; Slovenia; United Kingdom; and SSM: scheduled stop missing.

Concerning the ‘subcategories of animal welfare-related infringements,’ compared with those of 2011, overcrowding issues were almost three times more likely in 2009 (OR = 2.83, 95%CI = 1.54–5.26, *p* = 0.0009) and 2.6 times more likely in 2010 (OR = 2.62, 95%CI = 1.42–4.84, *p* = 0.002). A similar trend was also noticed for the presence of dead animals in the vehicles, which was 3.5 times more likely in 2009 than in 2011 (OR = 3.46, 95%CI = 1.03–12.27, *p* = 0.046). By contrast, the frequency of infringements that were related to the mixing of animals increased 10 times from 2009 to 2012 (OR = 10.16, 95%CI = 1.83–19.16, *p* = 0.031). Similarly, the probability of issues that were related to the presence of injured animals showed a six-fold increase from 2010 to 2013 (OR = 6.60, 95%CI = 1.80–25.81, *p* = 0.005; Figure 3a). A fluctuating trend over the years was found for the frequency of vehicles that were fined for missing a scheduled stop (MSS), which was far higher in 2011 than in 2009 (OR = 58.26, 95%CI = 10.67–1104.71, *p* = 0.0002). However, after 2011, this trend decreased, with 2012 and 2013 displaying, respectively, a 27- (OR = 27.75, 95%CI = 4.66–544.09, *p* = 0.003) and a 15-fold increase (OR = 15.59, 95%CI = 1.99–334.71, *p* = 0.022) in the frequency of vehicles that were fined for SSM when compared to that of 2009 (Figure 3a). MSS infringements were found, in particular, in vehicles from Belgium/Netherlands (compared with vehicles from Italy, OR= 146.70, 95%CI = 23.10–2926.57, *p* < 0.0001) and Spain (compared with vehicles from Italy, OR = 83.91, 95%CI = 13.90–1637.74, *p* < 0.0001; Figure 3b) that were mainly inspected in Northeast and Northwest Italy. Overcrowding-related infringements were associated with species (*p* = 0.037), with vehicles that were transporting sheep and goats (OR = 8.99, 95%CI = 1.26–63.86, *p* = 0.028) and pigs (OR = 10.94, 95%CI = 1.58–75.26, *p* = 0.015) being more likely to incur this type of penalty compared with “other species” (Figure 3c). Overcrowding in pigs was more frequently uncovered in fined vehicles that were coming from Italy (OR = 40.25, 95%CI = 4.33–1067.86, *p* = 0.005), Belgium, and the Netherlands (OR = 16.60, 95%CI = 1.97–403.24, *p* = 0.027) compared with vehicles from Spain (Figure 3d).

Concerning the subcategories that were related to the vehicle, compared with 2011, the probability of finding vehicles that were lacking in equipment for animal inspection had risen threefold by 2012 (OR = 3.25, 95%CI = 1.54–7.11, *p* = 0.002) and five-fold by 2013 (OR = 5.20, 95%CI = 1.43–16.97, *p* = 0.008). This infringement was also strongly associated with transported animal species. Indeed, vehicles that were transporting poultry were 9.5 times more likely to be sanctioned for this infringement than vehicles that were transporting other species (OR = 9.54, 95%CI = 2.38–49.82, *p* = 0.003) (Figure 3e).

Document-related infringements were more frequently noticed in vehicles that were transporting Equidae and other species, including dogs and cats (Figure 3f). Vehicles that were transporting “other species” were almost five times more likely than those transporting cattle to incur fines for the lack or incompleteness of veterinary documents (OR = 4.95, 95%CI = 2.02–12.05, *p* = 0.0004) and for the absence of the mark that indicated the presence of live animals (OR = 20.86, 95%CI = 3.34–147.30, *p* = 0.002). Similarly, in comparison with vehicles that were transporting cattle, vehicles that were transporting Equidae were three times more likely to incur penalties for the lack or incompleteness of veterinary documents (OR = 3.10, 95%CI = 1.71–5.60, *p* = 0.0002) and for the absence of the mark that indicated the presence of live animals (OR = 6.59, 95%CI = 2.01–24.86, *p* = 0.003).

### 3.4. Multiple Correspondence Analysis

**Figure 4:**
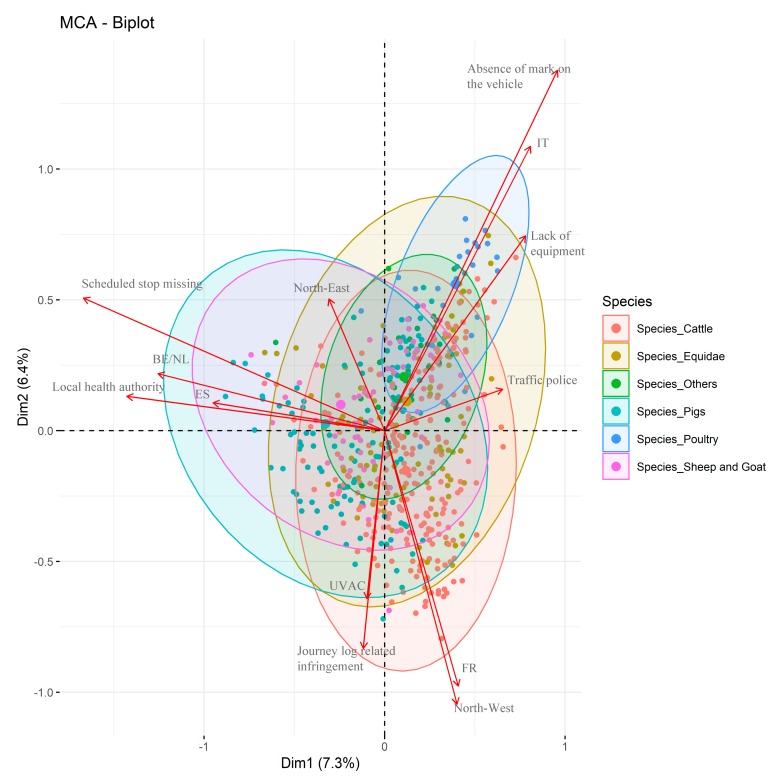
Multiple correspondence analysis biplot with the infringements distinguished on the basis of the transported animal species and the vectors representing the variables with the greatest effect on the two new dimensions (Dim1 and Dim2).

The top ten new dimensions that were identified by the MCA explained 47.2% of the total variance. The two dimensions that explained the highest variance percentages were Dimension 1 (Dim1) and Dimension 2 (Dim2), accounting for 7.3% and 6.4% of the total variance, respectively (Figure S1 and Figure 4). The contribution of the variable categories (in %) to the definition of the dimensions are reported in Supplementary Table S1. Dim1 mainly differentiated the infringements that were related to vehicles that were transporting poultry from the ones concerning the transport of pigs (Figure 4). Indeed, the variables that contributed the most to Dim1 were infringements that were related to missing a scheduled stop (16.97), local health authority (15.80) and traffic police (6.80) as supervisory bodies, Belgium/Netherlands (9.28) and Italy (6.54) as countries of dispatch, and pigs (6.74) as species. On the other hand, the variables that characterized Dim2 helped differentiate infringements that were linked to the transport of poultry from those reported for vehicles that were transporting cattle. Dim2 was indeed determined by the variables of the Northwest as place of inspection (14.27), Italy (13.61) and France (12.27) as dispatch countries, and infringements that concerned the journey log (9.80), poultry (5.89) and cattle (4.13) as transported animals. Dim 3 was mainly defined by Hungary/Romania as a country of dispatch (16.45), the UVAC as a supervisory body (15.94), and transported animals that belonged to other species (15.28). The center as an inspection place (19.50), infringements due to insufficient food (13.59), and unavailable or non-functioning drinking systems (9.16), together with sheep and goats as transported animals (7.55), were the variables that mostly affected Dim4. Dim5 was determined by Equidae (14.12) and poultry (6.94) as transported species, infringements caused by a lack of equipment for inspecting animals (13.61) and the absence on the vehicle of the mark that indicated the presence of live animals (13.25).

## 4. Discussion

This study analyzed the type of infringements that were uncovered during on-road inspections and recorded in the Italian Health Ministry reports from 2009 to 2013. Associations between the type of infringement (related to the welfare of the transported animals, vehicles and accompanying documents), year, transported species, the place of inspection, and competent authorities were identified. Based on our in-depth analysis of the infringements, it was possible to identify which types of animal movements (i.e., a particular species on a particular route) were most likely to be non-compliant with EC 1/2005, i.e., where more controls and enforcements of EC 1/2005 should be proposed. Furthermore, from our results, it was evident that while the chances of finding a transporter with improper documents or vehicle reduced over the period, the probability of an animal-welfare issue did not. Our findings also confirm that when traffic police were supported by official veterinarians during on-road inspections, greater emphasis was placed on the welfare of the transported animals. Consequently, the MoU seemed to be of great value for safeguarding the welfare of the transported animals, and such inspections should be strongly implemented in Italy and other member states. The results therefore help to propose recommendations on how to improve on-road inspections in Europe. This is the first manuscript that gives statistical evidence on live animal transportation in Italy after the enforcement of 1/2005, and such evidence could be useful for implementing the current regulation.

Our results highlight specific trends among fined vehicles. The distribution of the infringements among the transported species and the place of on-road inspection reflects the trade in live animals that has been occurring in the Italian livestock industry. It is indeed not surprising that the majority of the vehicles that were fined were transporting cattle, were coming from France, and were inspected in Northwest Italy, as the Italian beef industry relies on the importation of young bulls and heifers to complete the fattening phase, mainly in the north of Italy [20]. Similarly, it is well known that a large number of horses are imported to be slaughtered in the south of Italy [21], where horse meat is a traditional dish [22]. In 2012 alone, 36,465 horses were transported from one EU member state to another to an abattoir, and the vast majority of those horses were destined for Italy [21]. It has already been pointed out that there is considerable demand in Italy for fresh, local horsemeat, which could be the driving factor for the high demand for live horses as opposed to carcasses [23]. Moreover, the same study highlighted that processed meat from horses that originate in other countries but are slaughtered and processed locally can be stamped as produce of Italy [23]. The MCA demonstrated that the import of pigs was mainly from Netherlands and Belgium, as well as from Spain. This was expected because Spain is known to export fatteners. Belgium, Denmark, Germany and the Netherlands are major production areas of fattened pigs for slaughter, and the import of live pigs in Italy grew from 2009 to 2013 [24]. Among EU countries, Italy is one of the biggest importers of live pigs [25], which may be imported as young pigs to be fattened or as fattened pigs to be slaughtered for the production of high-quality dry cured hams, salami, and sausages. Finally, we confirmed that there was a high number of puppies that were coming illegally from the East of Europe [26]. The latter transporters were mainly fined due to a lack of veterinary certifications, as the animals were often being smuggled into Italy and were luckily often identified by the veterinary service that was working in Northeast Italy while coming from Eastern European countries [27]. The importation of puppies is not only a welfare concern due to the poor transport conditions they undergo [28] but also a huge biosecurity risk because the animals often do not have either veterinary certifications or mandatory vaccinations [29]. The higher number of vehicles that were transporting Equidae and other species with irregular documentation, in particular missing, incomplete, or non-compliant veterinary certificates, is a matter of concern for public health, as transport has already been identified as a risk factor for new diseases in companion animals and horses in Europe [30]. Overall, it is very important that official veterinarians enforce EC 1/2005, in particular at the borders, and it is a good sign that infringements that were related to accompanying documents reduced over the period investigated.

Infringements that were related to animal welfare did not decrease over the studied period, confirming the European Union’s concern [17]. However, our data show that the trend of animal-welfare infringements by year was not linear, with 2011 being very different from the others with a higher percentage of AW-related issues, in particular SSM (Figure 3a). This was probably due to a particular emphasis on this type of welfare that was issue by one of the authorities working in Northwest Italy. This was already noted in the Italian Ministry report for 2011 [31], which highlighted the need for a more standardized protocol of how to conduct on-road inspections within and among European countries as an important implementation of EC 1/2005. The European Union report [17] pointed out that member states should continue their efforts to provide harmonized data on transport inspections and infringement levels to the commission. Our analysis is the first to have been carried out in Italy, and a similar approach should be recommended to other member states. Interestingly, our results showed an agreement with the competences and roles of the different supervisory bodies. Vehicles were more likely to incur animal welfare-related penalties when they were inspected by supervisory bodies that were involved in the control of animals’ health and welfare, such as the local health authority and the veterinary service. In particular, it is worth mentioning that on-road inspections seemed to focus more on the welfare status of the transported animals when traffic police worked in synergy with the veterinary service. The role of official veterinarians (OVs) is crucial for safeguarding animal welfare during transport. OVs should support not only traffic police during on-road inspections but also veterinarians completing health certificates. OVs should also provide more training for transporters, farmers and drivers to help them recognize animals’ unfitness for transport [17].

Our results suggest that there was an association between infringement and transported species, in line with the literature. Recent research has suggested that the adverse effects of transportation on welfare vary by animal type. For example, mature or fat cattle (>500 kg) that were transported more than 400 km for slaughter had few quantifiable welfare issues (shrink, death, lameness, becoming non-ambulatory) compared to calves, feeders, and culls [32,33]. Cull cattle were found to be at the greatest risk of poor welfare during long-haul transportation because they had the greatest probability of becoming lame at the time of loading and unloading and being declared non-ambulatory or dead at the end of the journey compared to calves and feeders [33]. Consequently, it is crucial that vehicles that are transporting cattle over long distances do not skip mandatory stops at control posts. Our results suggest that infringements involving animal-welfare issues were more often associated with vehicles that were transporting pigs and sheep and goats. Vehicles that were transporting pigs were often fined for missing a scheduled stop and for overcrowding, in particular when coming from Italy, Belgium and Netherlands. Despite the fact that the mortality of pigs during transport has decreased in Europe, many studies, including ours, have suggested that current transport conditions of pigs are not effective at ensuring the welfare of pigs during long journeys across member states [13,34]. Vehicles that were transporting sheep and goats were more likely to show issues due to overcrowding, insufficient food, and unavailable or non-functioning drinking systems. Consequently, it is not surprising that those species were already found at higher risk of poor welfare when analyzing vehicles that were stopping at a control post in Southern Italy [1]. Our results of the MCA analysis indicated an increased probability of vehicles that were transporting horses to incur fines that were related to the lack of the mark that indicated the presence of live animals, to missing or improper drinking systems, and to the absence of a ventilation system and equipment for the inspection of animals. The poor transport conditions of horses that were designated for slaughter was denounced recently in a report published by Humane Society International, including a trade in unbroken horses that were travelling illegally over long distances [35]. The infringements that are associated with the transport of poultry were also characterized by higher proportions of issues that were related to the vehicle (a lack of equipment for the inspection of the animals and the mark that indicated the presence of live animals, in particular), whilst they were not associated with increased probabilities of incurring animal welfare-related fines. This may be due to the fact that animal-welfare surveys are extremely difficult, in particular when dealing with the inspection of higher tiers during transport. Considering that the number of consignments of poultry has shown a steady increase in the last ten years [17], an plan of how to conduct on-road inspection for this species is needed.

On-road inspections should be intensified on particular routes. Fined vehicles that were transporting poultry were mainly from Italy, whilst those transporting pigs came not only from Italy but also from Spain, Belgium/Netherlands, and France. Of particular interest is the evidence that the infringements that were associated with the transport of pigs from Spain and Belgium/Netherlands had an increased frequency of failed scheduled stops during transport, while fined vehicles that were transporting pigs from France showed more frequent journey log-related infringements. Similarly, fined vehicles that were transporting cattle that incurred journey log-related infringements were mainly inspected by the UVAC and were more frequently coming from France and inspected in Northwest Italy. Sheep and goats were mainly slaughtered in the center and south of Italy and in other Southern European regions (Spain, France, and Greece), where sheep and goat production has always played important economic, environmental and sociological roles [36]. Therefore, it was not surprising that vehicles that were transporting these species were more likely to be fined while transiting in the center of Italy. One of the most upsetting results was that the same vehicle was often fined for more than one reason, with the maximal numbers of infringements being nine, and the same company was often fined several times for the same reason. In the European Parliamentary Research Service (EPRS) (2018), it was recommended that in the case of ‘repeated or serious infringements,’ the penalty should be the suspension or withdrawal of the transporter’s authorization or certificate of approval for the means of transport concerned, or the temporary prohibition, for the transporter or means of transport concerned, from transporting animals in the country. In the case of multiple infringements, transporters should also lose their certificate of competence and be forced to re-attend a mandatory course. The enforcement of the regulation, the standardized interpretation of the requirements and relative intra-union penalties, and the implementation of controls by member states were requested in the European Commission reports [17,37]. However, the authors would like to suggest that an important implementation for safeguarding animal welfare during transport and reducing the number of infringements per means of transport would be providing training for the sector on both proper welfare practices and on what regulations mean.

This study was limited by the fact that we used a published dataset and we were not involved in the data collection. The ministry reports did not specify how many on-road inspections were conducted each year and whether or not the journey was within or longer than eight hours. Our analysis was therefore based only on transporters that were found to be in non-compliance with one or more articles of EC 1/2005. Given that the dataset therefore contained no records for transporters who did not receive a fine, we were unable to quantify the real frequency of the infringements and the real risk, as it was of course not possible to use having or not having received a fine as a dummy binary outcome. However, the combined use of ordinal regression and multivariate analyses gave us a good idea of how the different types of infringements varied over the examined period, the animal species transported, the supervisory body and the country of dispatch. The lack of information that was related to the type of journey (short or long) made it impossible for us to analyze the type of infringements based on the duration of the journey, which is a well-known risk factor for transport-related diseases [13,38,39]. The latter information should be added to the reports from member states in future. Finally, the present study considered the data up to the year 2013, as the method of detecting fines changed in 2014. Notwithstanding these limitations, this manuscript suggests a method for analyzing transport-related infringement after the enforcement of EC 1/2005, and we hope that it may be of inspiration for other scientists to start analyzing transport-related infringements that are issued in their own countries to fill the gap in the literature of which the European Parliament has complained [17]. This manuscript also highlights the important role of police officers and OVs during on-road inspections and how their discretion in deciding between sanctions and warnings should also be investigated for policy-implementation purposes [40].

## 5. Conclusions

There was a difference of the type of infringements that were uncovered (animal welfare-, vehicle- or document-related) depending on the competent authorities that were performing the road inspections, species transported, and country of dispatch. Over the years, there was only an improvement in accompanying documentation, while animal welfare-related infringements remained stable. The results underline the importance of on-road inspections for the identification of animal-welfare issues, in particular when road police and veterinarians work together. This type of road inspection should be intensified to enhance animal welfare during transportation. Traffic police officers should be trained in understanding their crucial role in enforcing EC 1/2005 and be motivated to focus efforts on conducting on-road inspections. Appropriate workshops to educate transporters and drivers on how to manage transport in an animal welfare-friendly manner seems to still be needed in order to reduce the number of infringement that are related to animal welfare. A standardized method on how to conduct on-road inspections should be implemented among competent authorities and member states so as to facilitate comparisons and safeguard animal welfare during transportation in Europe. Future studies should assess, in a more precise way, how the incidence of the various violations has been evolving since 2014, and they should identify a future strategy to limit animal transport-related issues.

## Figures and Tables

**Table 1:** Categories and subcategories of the infringements. Commercial vehicles that were transporting animals were sanctioned for failure to comply with one of more articles of Council Regulation (EC) 1/2005. The infringements that were recorded during the inspections were divided into three main categories: animal welfare (AW), vehicle (V), and accompanying documents (D).

Subcategory	Definition/Typology	Example
**Animal Welfare (AW)**		
Dead	Animals dead during transport	Rabbits dead due to insufficient air exchange
Improperly tied	Animals improperly tied during transport	Calves unable to lie down because they were tethered on a too short rope
Injured	Animals injured during transport	Some pigs with skin lesions
Mixing different sexes or ages	Animals not properly divided during transport	Stallions not separated from mares
Overcrowding	Load density not respected	Excessive load density
Unfit	Animals unfit for transport	Calves less than 10 days old. Horses with fever. Cattle with severe lameness
Scheduled Stop Missing (SSM)	Failure to make the scheduled stop during transport	Failure to make the scheduled stop at the resting place scheduled
**Vehicle (V)**		
Bedding	Not present (NP)	Bedding absent on board
	Not sufficient (NS)	Poor bedding
Deck	Height not sufficient	Height of the load compartment less than 75 cm above the withers
Dirty	Insufficient degree of cleaning	Feces and urine leaked from the vehicle
Drinking system	Not present (NP)	Absence of availability of drinking troughs
	Out of order (OO)	Watering device not working
	Lack of water (LW)	Watering device insufficient
	Inappropriate for the species (INA)	Unsuitable watering system
Feed	Food not sufficient for the number of animals transported	Insufficient amount of food
Lack of equipment	Vehicle not equipped with all the necessary devices to carry out an adequate on-road inspection	No ladder for animal inspection
Lighting system	Not sufficient (NS)	Insufficient lighting inside the trailer
Mechanical ventilation system	Not present (NP)	No ventilation system
	Not sufficient (NS)	Subjects dead due to insufficient air exchange
	Out of order (OO)	Ventilation system not working
Navigation	Satellite navigation system not present or out of order	Vehicle not equipped with satellite navigation system
Partitions	Not present (NP)	Absence of individual dividers
	Present but not set in place or unsuitable (P)	Internal partitions without full walls and with unsuitable closures
Ramp	Ramp not suitable for animal unloading and loading	Unloading ramp without side protection
Temperature	Temperature recording system not working properly	Temperature printouts not available
**Accompanying Documents (D)**		
Infringement of legislation	Travel not in accordance with European regulations	Non-compliance of the vehicle with Annex I, Chapter II, EC Regulation 1/2005
Journey log	Excessive journey time (EJT)	Infringement of maximum transport times
	Non-compliant (NC) (Expected duration missing, time of departure missing, resting places missing, resting places irregular, unrealistic expected duration, plan missing)	Journey log not compliant because irregularly compiled
	Not present (NP)	Absence of the journey log
	Unbound	Journey log not bound
	Unsigned	Journey log not signed
	Unstamped	Journey log not stamped
Lack of emergency plan	Emergency plan not present or non-compliant	Lack of phone numbers of OVs and slaughterhouses to call in case of emergency
Watering/feeding plan	Watering/feeding plan not present or non-compliant	Lack of written instructions for the administration of water and food during the journey and possible treatment in case of need
Type-approval certificate	Type-approval certificate not present or non-compliant	Absence of vehicle type-approval certificate
Transporter authorization	Transporter authorization or certificate of competence not present or non-compliant	Absence of type 2 transporter authorization Absence of certificate of competence
Vehicle marked	Indication of live animals not present or non-compliant	Vehicle without the mark that indicates the presence of live animals
Veterinary certificate	Irregular or incomplete	Absence of transport authorization
	Irregular passport	Calves without health certificate and passport

**Table 2:** Animal welfare-related infringement severity scores.

Score	Categories of infringement	Example
1	One infringement for D or V	Three watering devices without water.
2	One infringement on AW, or a double infringement D + V	Cages unsuitable for stool containment and absence of transporter authorization.
3	Double or triple infringement: AW + V, AW + D, AW + V + D	Journey log filled out incorrectly; pigs stressed and injured due to insufficient space; insufficient water and food devices.

AW: animal welfare; V: vehicle; and D: documents.

**Table 3:** Descriptive statistics of the infringements that were uncovered during live animal transportation as recorded in the Italian Health Ministry reports on the infringements that were written in compliance with Article 27 of EC 1/2005 from 2009 to 2013. The number of vehicles that were found not to be complying with one or more articles of EC 1/2005 according to year, species, the country of dispatch, the country of destination, supervisory body, and the place of inspection.

Factor Categories	Number	Percentage (%)
**Year:**		
2009	161/985	16.35
2010	154/985	15.63
2011	292/985	29.64
2012	201/985	20.41
2013	177/985	17.97
**Animal Species**		
Cattle	437/979	44.64
Pigs	201/979	20.53
Equidae	151/979	15.42
Sheep and Goat	107/979	10.93
Poultry	45/979	4.60
Others (dogs, cats, fish, rabbits, eels, hares, penguins, pigeons, red-legged partridge, reptiles, rodents, and squirrels)	38/979	3.88
Missing data	6/979	0.61
**Country of Dispatch**		
France	278/968	28.72
Italy	250/968	25.83
Spain	132/968	13.64
Belgium/the Netherlands	112/968	11.57
Hungary/Romania	80/968	8.26
Germany/Poland	73/968	7.54
Others (CHN, CZ, DK, IRL, LT, SK, SVN, UK)	43/968	4.44
Missing data	17/968	1.73
**Country of Destination**		
Italy	884/964	91.70
Greece	44/964	4.56
Others (AT, BG, DE, ES, FR, HU, PL, RO)	36/964	3.73
Missing data	21/964	2.13
**Supervisory Body**		
Traffic police	402/985	40.81
Local health authority (LHA)	249/985	25.28
Veterinary officers for compliance with EU requirements (UVAC)	193/985	19.59
Traffic police and veterinary service (UVAC or LHA)	113/985	11.47
Others (state forestry corps, Carabinieri, customs corps, border inspection posts)	28/985	2.84
**Place**		
Northeast	434/978	44.38
Northwest	284/978	29.04
Center	196/978	20.04
South	64/978	6.54
Missing data	7/978	0.71

CHN: China; CZ: Czech Republic; DK: Denmark; IRL: Ireland; LT: Lithuania; SK: Slovakia; SVN: Slovenia; UK: United Kingdom; AT: Austria; BG: Bulgaria; DE: Germany; ES: Spain; FR: France; HU: Hungary; PL: Poland; and RO: Romania.

**Table 4:** Number of infringements that were related to animal welfare (AW), vehicle (V) and documents (D) or to a combination of two or three of them.

Type	Number	Percentage (%)
D	339/985	34.42
AW	276/985	28.02
V	126/985	12.79
V + D	78/985	7.92
AW + V	58/985	5.89
AW + D	54/985	5.48
AW + V + D	54/985	5.48

Legend. D: Documents; AW: Animal Welfare; V: Vehicle.

## Supplementary Materials

The following are available online at https://www.mdpi.com/2076-2615/10/2/356/s1, Figure S1: The percentage of total variance explained by the first ten dimensions identified by the Multiple Correspondence Analysis. Table S1: The contribution of the variable categories (in %) to the definition of the dimensions of the Multiple Correspondence Analysis.
