# Supplementary material for: Protection of Animals during Transport: Analysis of the Infringements Reported from 2009 to 2013 during On-Road Inspections in Italy
Source: Animals (Basel). 2020 Feb 22;10(2):356. doi: 10.3390/ani10020356 (PMC7070427; doi:10.3390/ani10020356)
Supplement: Supplementary file 1 [file animals-10-00356-s001.pdf]

Supplementary Materials

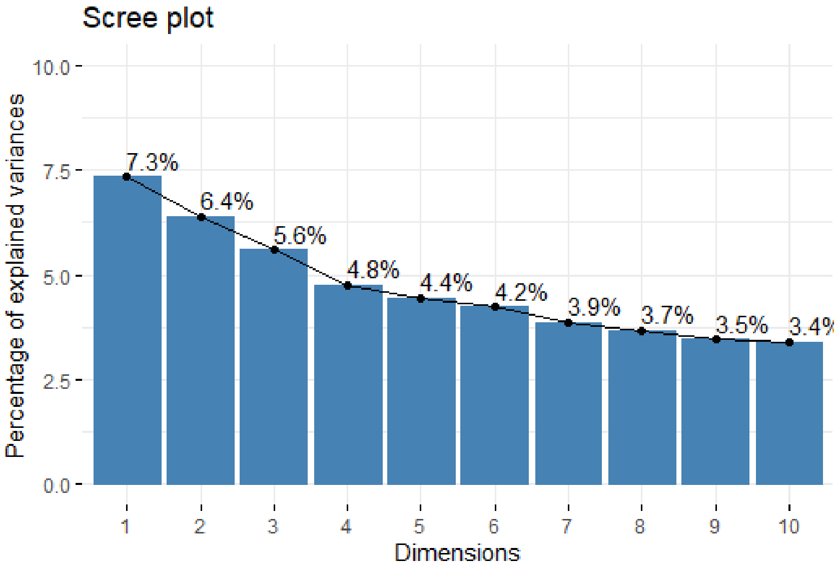

Figure S1. The percentage of total variance explained by the first ten dimensions identified by the Multiple Correspondence Analysis.

Table S1. The contribution of the variable categories (in %) to the definition of the dimensions of the Multiple Correspondence Analysis.

Variables	Dimension 1	Dimension 2	Dimension 3	Dimension 4	Dimension 5
Cattle	1.88	4.13	0.33	3.53	1.32
Equidae	0.71	0.76	0.16	0.88	14.12
Other species	0.14	0.67	15.28	0.47	0.05
Pigs	6.74	0.06	4.01	0.18	0.47
Poultry	2.18	5.89	0.20	0.11	6.94
Sheep and Goat	1.93	0.43	5.58	7.55	2.23
Belgium/Netherland	9.28	0.09	5.22	0.02	1.17
Deutschland/Poland	0.01	0.12	5.56	0.00	0.86
Spain	4.74	0.07	0.35	1.96	0.29
France	1.87	12.27	1.46	2.12	0.20
Hungary/Romania	0.12	0.24	16.45	2.73	1.10
Italy	6.54	13.61	1.44	0.00	0.16
Other countries of dispatch	0.11	1.28	0.00	1.46	0.15
Local Health Authority	15.80	0.55	0.83	0.00	0.00
Other supervisory bodies	0.36	0.56	0.09	1.61	0.07
Traffic police	6.80	0.46	2.30	0.56	0.31
Traffic police and veterinary service	1.32	0.05	1.46	0.19	4.00
Veterinary Offices for Compliance with EU Requirements (UVAC)	0.07	3.68	15.94	0.15	0.35
Centre	0.73	2.51	0.36	19.50	3.96
North-East	1.66	5.08	1.13	4.17	5.55
North-West	1.83	14.27	1.17	1.86	0.41
South	0.97	0.39	0.34	0.42	1.60
Overcrowding_0	0.04	0.20	0.91	0.63	0.03
Overcrowding_1	0.20	0.97	4.46	3.10	0.13
Unfit_0	0.03	0.07	0.00	0.18	0.02
Unfit_1	0.60	1.34	0.00	3.29	0.35
Stop missing_0	3.15	0.33	0.06	0.51	0.19
Stop missing_1	16.97	1.80	0.34	2.74	1.05
Drinking_0	0.02	0.21	0.05	0.92	0.76

Drinking_1	0.17	2.09	0.49	9.16	7.62
Ventilation_0	0.01	0.01	0.03	0.09	0.17
Ventilation_1	0.28	0.37	1.02	3.03	5.48
Deck_0	0.09	0.12	0.02	0.03	0.01
Deck_1	2.03	2.73	0.37	0.63	0.32
Lack_0	0.22	0.22	0.04	0.12	1.36
Lack_1	2.15	2.25	0.36	1.23	13.61
Feed_0	0.00	0.00	0.03	0.23	0.01
Feed_1	0.14	0.07	1.82	13.59	0.59
Dirty_0	0.03	0.01	0.00	0.00	0.03
Dirty_1	1.19	0.24	0.09	0.15	1.21
Temperature_0	0.01	0.04	0.05	0.14	0.10
Temperature_1	0.18	1.08	1.16	3.39	2.53
Veterinary_0	0.22	0.08	0.30	0.06	0.00
Veterinary_1	1.49	0.53	2.00	0.39	0.00
Journey log_0	0.08	4.53	0.42	1.40	0.55
Journey log_1	0.17	9.80	0.92	3.02	1.19
Vehicle marked_0	0.04	0.10	0.01	0.05	0.47
Vehicle marked_1	1.23	2.92	0.17	1.41	13.25
Type approval certificate_0	0.11	0.02	0.08	0.00	0.14
Type approval certificate_1	1.65	0.34	1.13	0.05	1.98
Transporter authorisation_0	0.12	0.02	0.26	0.06	0.00
Transporter authorisation_1	1.40	0.18	3.08	0.73	0.00
Infringement of legislation_0	0.00	0.00	0.01	0.00	0.01
Infringement of legislation_1	0.18	0.16	0.61	0.16	1.08
